# Culture Conditions Affect Expression of DUX4 in FSHD Myoblasts

**DOI:** 10.3390/molecules20058304

**Published:** 2015-05-08

**Authors:** Sachchida Nand Pandey, Hunain Khawaja, Yi-Wen Chen

**Affiliations:** 1 Research Center for Genetic Medicine, Children’s National Medical Center, Washington, DC 20010, USA; E-Mails: spandey@childrensnational.org (S.N.P.); HKhawaja@childrensnational.org (H.K.); 2 Department of Integrative Systems Biology and Department of Pediatrics, George Washington University, Washington, DC 20037, USA

**Keywords:** DUX4, Superscript, FSHD, dexamethasone, KOSR

## Abstract

Facioscapulohumeral muscular dystrophy (FSHD) is believed to be caused by aberrant expression of double homeobox 4 (DUX4) due to epigenetic changes of the D4Z4 region at chromosome 4q35. Detecting DUX4 is challenging due to its stochastic expression pattern and low transcription level. In this study, we examined different cDNA synthesis strategies and the sensitivity for DUX4 detection. In addition, we investigated the effects of dexamethasone and knockout serum replacement (KOSR) on DUX4 expression in culture. Our data showed that DUX4 was consistently detected in cDNA samples synthesized using Superscript III. The sensitivity of DUX4 detection was higher in the samples synthesized using oligo(dT) primers compared to random hexamers. Adding dexamethasone to the culture media significantly suppressed DUX4 expression in immortalized (1.3 fold, *p* < 0.01) and primary (4.7 fold, *p* < 0.01) FSHD myoblasts, respectively. Culture medium with KOSR increased DUX4 expression and the response is concentration dependent. The findings suggest that detection strategies and culture conditions should be carefully considered when studying DUX4 in cultured cells.

## 1. Introduction

Facioscapulohumeral muscular dystrophy (FSHD) is a dominant muscular dystrophy, with a prevalence of 1:20,000, and the third most common type of muscular dystrophy [1]. The disease is characterized by a progressive weakness and atrophy of the facial, scapular, and humeral muscles followed by weakness of muscles of the lower extremities [2]. While clinically indistinguishable, FSHD is sub-classified into FSHD1 and FSHD2 depending on the genetic causes. FSHD1 is genetically linked to contractions of the D4Z4 repeat array at chromosome 4q35 and affects approximately 95% of the patients. In FSHD1, the D4Z4 array is contracted from 11–150 repeat units in unaffected individuals to 1–10 repeat units in the patients [3,4,5]. Each D4Z4 repeat contains a double homeobox protein 4 (DUX4) which is cytotoxic when ectopically expressed, as shown by both *in vivo* and *ex vivo* studies [6,7,8,9,10]. Previous studies showed that a combination of two genomic features is required to cause FSHD. First, the contraction of the D4Z4 repeats, which leads to DNA hypomethylation of the D4Z4 region and allows DUX4 mRNA to be transcribed [11]. Second, an intact polyadenylation signal in the region distal to the last repeat of D4Z4, which allows the *DUX4* transcripts from the last D4Z4 repeat to be polyadenylated, thus stable for protein translation. The combination leads to the aberrant expression of DUX4 and downstream molecular changes involved in FSHD [12,13,14,15]. This disease model was further supported by the identification of mutations in a gene named structural maintenance of chromosomes flexible hinge domain containing 1 (SMCHD1) in FSHD2, which encodes a chromatin modifier of D4Z4. Haploinsufficiency of the SMCHD1 coupled with the FSHD permissive allele was shown to be causative of the FSHD2 [16].

DUX4 expression in primary myoblasts from patients with FSHD has been showed to be stochastic. Previous studies report 1 in 1000 nuclei is DUX4 positive in proliferating FSHD myoblasts; and 1 in 200 nuclei during myoblast differentiating [17,18]. Due to the extremely low expression level of the DUX4, detection of DUX4 transcripts has been challenging. Several different DUX4 detection strategies have been reported, using different reverse transcriptases (RT) and primers [e.g. oligo(dT), random hexamers] for cDNA synthesis [13,19,20,21]. In some studies, DUX4 expression was amplified using nested PCR [17,22]. These strategies have not been compared side-by-side, and often fail to detect endogenously produced DUX4 in a different laboratory.

While DUX4 expresses in very few number of cells during proliferation, the expression level of DUX4 increases during differentiation. This observation suggests that the expression of DUX4 can be modulated by the physiological state of the cells and culture environment. Dexamethasone is a glucocorticoid which stimulates myoblasts proliferation and is often added in culture to promote cell growth [23]. KOSR is a serum replacement often used for culturing stem cells and was suggested to protect cells via its antioxidative property [24]. A recent study also reported higher DUX4 expression in differentiating FSHD myoblasts cultured in medium with 20% KOSR [19]. In this study, we cultured immortalized FSHD myoblasts in growth medium with or without dexamethasone and KOSR, respectively, to determine the effects of culture conditions on DUX4 expression. In addition, effects of different concentrations of KOSR on DUX4 expression in differentiating cells were determined. The purpose of this study is to identify a sensitive detection strategy for DUX4 transcripts and to determine how culture conditions affect DUX4 expression in cell culture.

## 2. Results and Discussion

**Figure 1:**
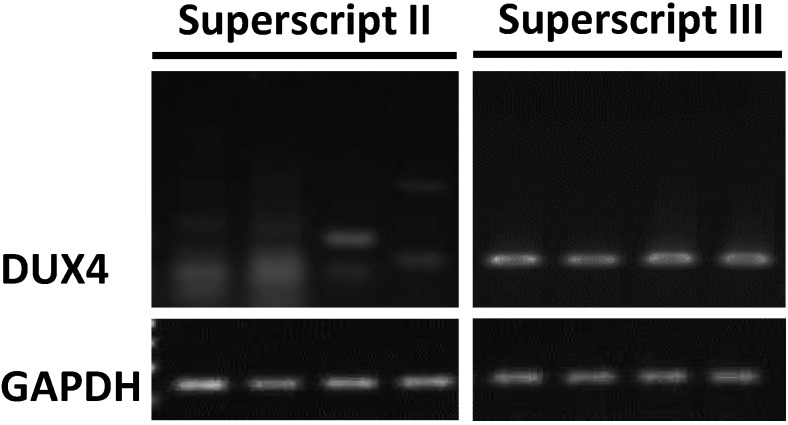
DUX4 was detected in cDNA synthesized using Superscript III but not Superscript II. Four independent cultures of immortalized FSHD myoblasts in growth medium with 15% KOSR (without dexamethasone) were examined for each group. cDNA samples were synthesized using either Superscript II (left panel) or Superscript III (right panel). Oligo(dT) primers were used for this experiment.

**Figure 2:**
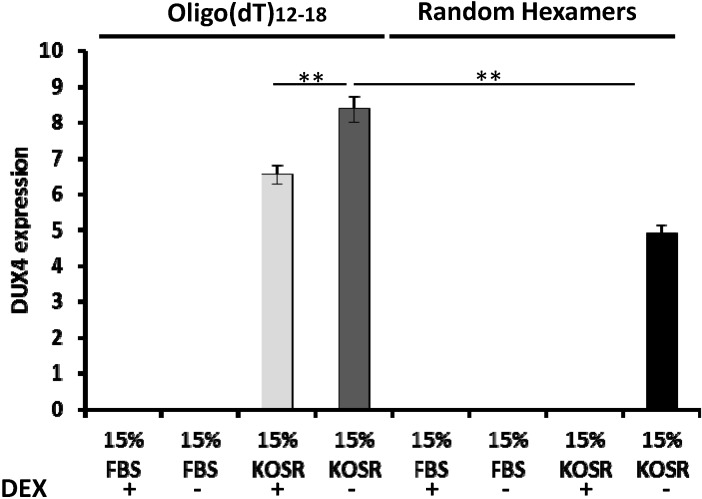
Higher sensitivity of DUX4 detection in cDNA samples synthesized using oligo(dT) primers compared to those synthesized using random hexamers. In addition, higher expression of DUX4 was detected in cells cultured in growth medium with 15% KOSR instead of 15% FBS. Adding dexamethasone reduced DUX4 expression. DEX, dexamethasone; FBS, fetal bovine serum; KOSR, knockout serum replacement; “**”: *p* < 0.01.

Detecting DXU4 expression in FSHD myoblasts has been very challenging, primarily due the stochastic expression and cytotoxic nature of DUX4, which lead to an extremely low number of cells expressing DUX4 in culture. Different sample preparation and RT conditions have been used and reported previously, which are listed in Supplementary Table S1. In order to identify the most effective method to detect DUX4 expression in FSHD myoblasts, we tested two most commonly used reverse transcriptases, Superscript II and Superscript III, and two types of primers, oligo(dT) and random hexamers. Our result showed that DUX4 expression was easily detected in FSHD myoblasts when the cDNA was synthesized using Superscript III. Whereas DUX4 expression was not detected in cDNA samples synthesized using Superscript II (Figure 1). In addition to the reverse transcriptases, we examined the effects of primers used for cDNA synthesis. Our data showed that higher DUX4 expression (1.7 fold, *p* < 0.01) was detected in cDNA samples synthesized using oligo(dT) primers compared to random hexamers (Figure 2).

### 2.1. KOSR and Dexamethasone in Culture Media Affect DUX4 Expression in FSHD Myoblasts

To determine the effects of KOSR and dexamethasone on DUX4 expression in culture, we cultured FSHD immortalized myoblasts in regular growth medium containing 15% FBS with dexamethasone, 15% FBS medium without dexamethasone, 15% KOSR with dexamethasone and 15% KOSR medium without dexamethasone. Our data showed that DUX4 expression was not detected in FSHD myoblasts cultured in growth medium containing FBS, either with or without dexamethasone. DUX4 expression was detected in FSHD myoblasts cultured in growth medium with KOSR (Figure 2). DUX4 expression in FSHD myoblasts cultured in KOSR medium without dexamethasone was 1.3 fold higher (*p* < 0.01) than cells cultured in KOSR medium with dexamethasone (Figure 2).

**Figure 3:**
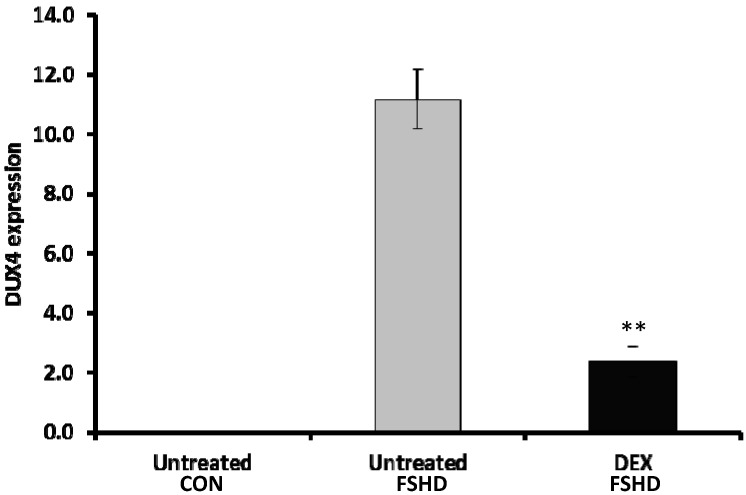
Dexamethasone reduced DUX4 expression in primary FSHD myoblasts. Control (*n* = 5) and FSHD (*n* = 5) myoblasts were cultured in growth medium with 15% KSOR, either with or without dexamethasone, respectively, for 3 days. DEX, dexamethasone; CON, control; “**”: *p* < 0.01.

To increase biological sample size, we further validated the effects of dexamethasone on DUX4 expression using 5 pairs of primary myoblasts. Primary FSHD myoblasts were cultured in growth medium with 15% KSOR (with or without dexamethasone) for 3 days, respectively. We observed a significant reduction in DUX4 expression (4.7 fold, *p* < 0.01) in dexamethasone-treated FSHD myoblasts in comparison to untreated FSHD myoblasts (Figure 3).

**Figure 4:**
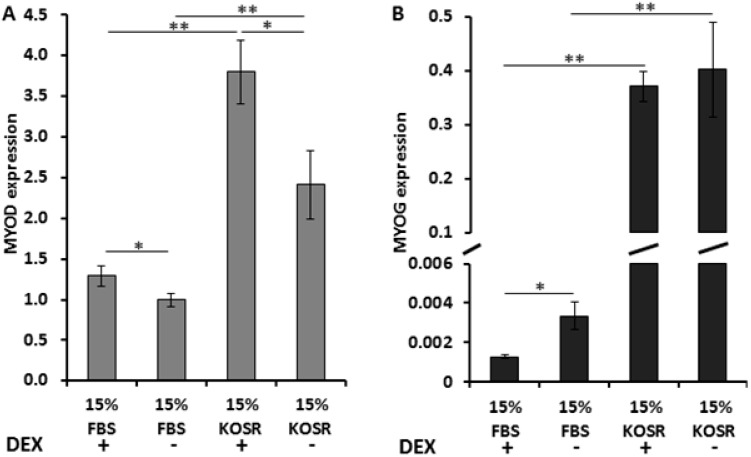
KOSR and dexamethasone affect MYOD and MYOG expressions in immortalized FSHD myoblasts. Expression level of MYOD (**A**) and MYOG (**B**) were affected differently by KOSR and dexamethasone. DEX, dexamethasone; FBS, fetal bovine serum; KOSR, knockout serum replacement; “*”: *p* < 0.05; “**”: *p* < 0.01.

KOSR containing differentiation medium has been reported to facilitate differentiation of FSHD myoblasts and increases DUX4 expression in myotubes [19]. Therefore, we examined expression level of two myogenic factors, myogenic differentiation antigen 1 (MYOD) and myogenin (MYOG), in the FSHD myoblasts cultured in media containing either FBS or KOSR, with or without dexamethasone, respectively. Expression of both MYOD and MYOG increased in myoblasts cultured in growth medium containing KOSR (Figure 4). Adding dexamethasone increased MYOD expression in the myoblasts cultured in medium containing FBS as well as in medium containing KOSR (Figure 4A). Adding dexamethasone did not significantly affect MYOG expression in myoblasts cultured in growth medium containing KOSR (Figure 4B). However, MYOG was decreased by dexamethasone in myoblasts cultured in growth medium containing FBS (Figure 4B).

### 2.2. Concentration-Dependent Increase of DUX4 in FSHD Myotubes Cultured in Differentiation Medium with KOSR

Horse serum at 2% concentration is commonly used in differentiation media. To determine whether KOSR promote differentiation and DUX4 expression more than HS, we replaced 2% HS with 2% KOSR in differentiation media. In addition, we determined how different concentrations (2%, 10%, 15% and 20%) of KOSR affected DUX4 expression in FSHD myotubes.

**Figure 5:**
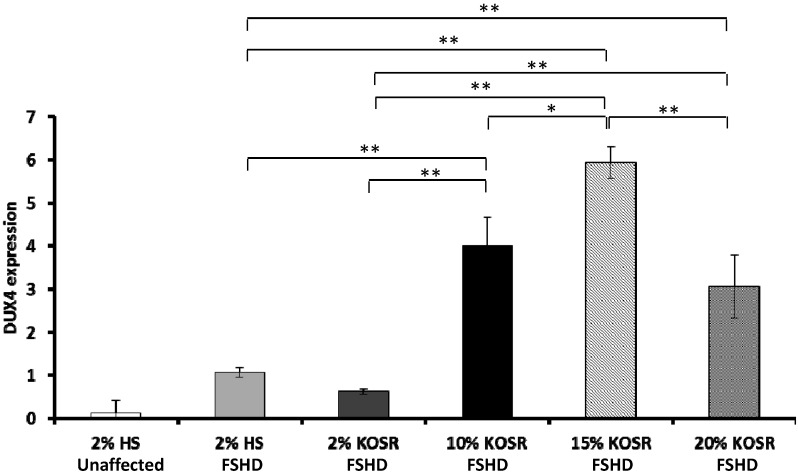
Concentration-dependent increase of DUX4 in differentiated FSHD myotubes in response to KOSR. Immortalized FSHD myoblasts were differentiated in media with 2% HS, 2% KOSR, 10% KOSR, 15% KOSR, and 20% KOSR, respectively. The mRNA expression of DUX4 were quantified using qRT-PCR. CON, control; * *p* < 0.05; ** *p* < 0.01.

Our results showed that DUX4 expression was not significantly higher in FSHD myotubes cultured in 2% KOSR, however it was increased in FSHD myotubes cultured in 10% KOSR (3.77 fold, *p* < 0.01), 15% KOSR (5.58 fold, *p* < 0.01), and 20% KOSR (2.87 fold, *p* < 0.01) compared to 2% HS (Figure 5). In addition, the expression was significantly different among the cultures with different concentrations of KOSR. DUX4 expression was the highest in myotubes cultured in differentiation medium with 15% KOSR (Figure 5).

### 2.3. Discussion

Ectopic expression of DUX4 in cells and animal models has been shown to induce cell apoptosis and is cytotoxic [6,9,10]. It is hypothesized that few DUX4-positive FSHD myoblasts are detected in culture because the expression of DUX4 is stochastic, and once the DUX4 is expressed in certain cells, the cells expressing DUX4 do not survive long. In this study, we examined different cDNA synthesis strategies to determine which strategy can most sensitively detect DUX4 expression in cultured FSHD myoblasts. In addition, we identified cell culture conditions that affected expression level of DUX4 in cell culture, including agents that increased and reduced DUX4 expression, respectively. The findings showed that, in addition to detecting DUX4, the expression of DUX4 can be significantly affected by the culture condition; therefore, the culture conditions need to be taken into consideration when planning an experiment using FSHD myoblasts.

Several different DUX4 detection strategies have been reported previously, including different reverse transcriptases (RT) and primers [e.g., oligo(dT), random hexamers] for cDNA synthesis [13,20,21]. Our results showed that Superscript III is more sensitive than Superscript II for detecting DUX4. This is likely due to that Superscript III is more sensitive for detecting RNAs expressing at low levels. In a previous study Superscript III, but not Superscript II, was shown to be one of the 5 RT enzymes with the highest sensitivity in detecting low copy RNA among 11 RT enzymes tested [25]. In addition to the RT enzymes, our data showed that oligo(dT) priming increased detection sensitivity compared to random hexamers priming. A previous study suggested that a higher coverage of transcripts were synthesized when oligo(dT) primers were used. In this study, only two out of 8 genes were efficiently detected in cDNA synthesized by hexamers, while all genes were efficiently detected in cDNA synthesized by oligo(dT) [26]. In our study, both priming methods allowed DUX4 detection but with different sensitivities. This may be due to that cDNA samples generated with random hexamers tend to carry sequence-specific mismatch errors, which can potentially reduce priming efficiency during PCR [27].

KOSR is an artificial serum replacement with a defined formula (Life Technologies, Frederick, MD, USA). It is often used for culturing stem cells and has been shown to have various cellular effects, including interfering with wnt-mediated cell differentiation, and protect stem cells from apoptosis via its anti-oxidative components [24,28]. Previous studies have reported that higher DUX4 expression is associated with increased myoblast differentiation [18,19]. In our study, myogenic factors, MYOD and MYOG were up-regulated in myoblasts cultured in growth medium with 15% KOSR (3 days in KOSR), which suggested that replacing 15% FBS with 15% KOSR in a typical growth medium might have initiated cell differentiation program. This finding suggested that the expression change of DUX4 may be in response to cell differentiation. In addition, KOSR may not be used to replace FBS in growth media for maintaining FSHD myoblasts. Meanwhile, other known effects of KOSR may explain the higher expression of DUX4 in the FSHD myoblasts. A previous study reported that suppressing wnt signaling activated DUX4 expression [19]. The wnt suppressive effects of KOSR may have increased DUX4 expression in this case. In addition, FSHD cells have been shown to be susceptible to oxidative stress [29]. KOSR contains antioxidant such as selenium and ascorbate, which have been shown to protect stem cells from apoptosis. FSHD myoblasts cultured in KOSR may have been better protected from oxidative stress and apoptosis, therefore higher number of DUX4-positive cells survived in culture.

Dexamethasone has been shown to suppress wnt signaling in epithelial and osteoblasts cells [30,31]. Whether it suppresses wnt signaling in myoblasts is not clear. In addition to effects on wnt signaling, dexamethasone has been shown to increase ROS generation and apoptosis in proliferating human rhabdomyosarcoma cells [32]. In this case, dexamethasone may potentially increase DUX4 expression. Our data showed lower expression of DUX4 when the FSHD myoblasts were cultured in media with dexamethasone. One explanation is that the myoblasts cultured in dexamethasone may die quicker due to oxidative stress and cell death signaling induced by dexamethasone, implicating a narrow window for DUX4 detection. Since adding dexamethasone is a common practice in cell culture, whether it is harmful to the DUX4 expressing cells need to be further investigated.

Our studies showed that reverse transcriptase enzymes and primers used for cDNA synthesis significantly affect the detection of DUX4 in FSHD myoblasts in culture. Superscript III and oligo(dT) are the best combination when synthesize cDNA for DUX4 detection. Differences in culture conditions including dexamethasone which are often added for improving cell proliferation and knockout serum replacement (KOSR) significantly affect the expression of DUX4. The addition of KOSR in culture increased DUX4 while dexamethasone reduced DUX4 detection. Understanding the molecular mechanisms underlying these observations may help decipher regulation of DUX4 expression in FSHD myoblasts.

## 3. Materials and Methods

### 3.1. Human Myoblasts Culture

Immortalized and primary myoblasts were obtained from the Senator Paul Wellstone Muscular Dystrophy Cooperative Research Center at Boston Biomedical Research Institute [33]. The patient myoblast cell line was derived from the biceps of a 42 year-old male with mild muscle weakness. The control myoblasts were derived from the patient’s 46 year-old brother without FSHD [33,34]. These cells were cultured as previously described [33,34,35]. Briefly, both the immortalized and primary myoblasts were cultured in LHCN medium with or without dexamethasone (140 nM/mL) (Sigma-Aldrich, St. Louis, MO, USA) [4:1 DMEM:Medium 199 supplemented with 15% characterized FBS (Hyclone, South Logan, UT, USA), 0.02 M HEPES (Sigma-Aldrich), 0.03 mg/mL ZnSO_4_ (Sigma), 1.4 mg/mL Vitamin B12 (Sigma-Aldrich), 2.5 ng/mL hepatocyte growth factor (Chemicon International, Temecula, CA, USA), 10 ng/mL basic fibroblast growth factor (Millipore, Billerica, MA, USA) 0.02 M HEPES, and (Life Technologies)] kat 37 °C, 5% CO_2_ for 6 days. For testing the effects of KOSR, 15% FBS in the LHCN medium were replaced with 15% KOSR (either with or without dexamethasone) for 3 days before the cells were collected for RNA isolation. Total RNA was isolated using mirVANA kit (Life Technologies). Quadruplicates were performed for all studies.

To determine the effects of KOSR concentrations on DUX4 expression in differentiating myoblasts, immortalized FSHD and unaffected myoblasts were cultured in LHCN medium without dexamethasone for 3 days. The LHCN medium was then replaced with differentiation medium. The differentiation medium consist of antibiotics/antimycotics 1%, L-glutamine 2 mM, sodium pyruvate 1 mM, HEPES 0.02 M and 2% HS or different concentrations of KOSR (2%, 10%, 15% and 20%). The myoblasts were differentiated for 7 days then harvested for RNA isolation using the mirVANA kit (Life Technologies).

### 3.2. The cDNA Synthesis and RT-PCR

RNA isolation, cDNA synthesis, semi-quantitative RT-PCR were performed as previously described [35]. Total RNA was isolated using mirVANA kit (Life Technologies) according to the manufacturer’s protocol. Three micrograms of total RNA was treated with DNase I (Promega, Madison, WI, USA) digestion by incubating at 37 °C for 30 min to remove genomic DNA contamination. The reaction was inactivated by solution provided in the kit (Promega) and heated for 10 min at 65 °C. The samples were purified using the RNeasy MinElute Cleanup Kit (Qiagen, Hilden, Germany) according to the manufacturer’s protocol. Subsequently, the 1 µg of RNA sample was reverse transcribed to cDNA using Superscript II and Superscript III (Life Technologies), respectively. Briefly, RNA samples were incubated at 65 °C for 5 min with 1 µL dNTPs (10 mM each) (New England Biolab, Ipswich, MA, USA) and 1 µL oligo(dT)_12–18_ (Life Technologies) or 1 µL of random hexamers (Life Technologies), respectively. Thereafter, a master mix of 4 µL 5X first strand buffer (Life Technologies), 1 µL DTT (Life Technologies), 1 µL RNasin (Promega) and either Superscript II or Superscript III (Life Technologies), respectively, were added in each reaction and incubated for 25 °C for 5 min, 50 °C for 1 h and 70 °C for 15 min. The 1 µL of RNase H (Life Technologies) was added to each reactions of cDNA synthesis and incubated for 37 °C for 20 min.

The cDNA was amplified using GoTaq green master Mix (Promega) using 1 µM of forward and reverse primers specific to each gene and 3 µL (60 ng) of cDNA template in a total volume of 20 µL. The thermal cycling conditions are 95 °C for 3 min, followed by 40 or 30 cycles of 95 °C for 10 s then 62 °C for 45 s for cDNA samples synthesized from immortalized and primary myoblasts, respectively. The samples were kept at 72 °C for 10 min at the end. Primer sequences used for human *DUX4* were (forward) 5'-CCCAGGTACCAGCAGACC-3' and (reverse) 5-TCCAGGAGATGTAACTCTAATCCA-3'. *Glyceraldehyde-3-phosphate dehydrogenase* (*GAPDH*) was used as internal control and the primers used were (forward) 5'-TTGTCAAGCTCATTTCCTGGTATG-3' and (reverse) 5'-GTGAGGGTCTCTCTCTTCCTCTTGT-3'. The primer sequences for a second internal control, *18S rRNA*, were (forward) 5'-ATTGCAATTATTCCCCATGAACG-3' and (reverse) 5'-CACTAAACCATCCAATCGGTAGTAGC-3'. PCR products were visualized in 2.0% agarose gel by electrophoresis. Densitometric analysis of EtBr-stained gel for DUX4 and internal control bands was performed using Image J software (NIH, Bethesda, MD, USA).

Real-time qRT-PCR was performed as previously described [12]. The thermal cycling condition was an initial 50 °C for 2 min, 95 °C for 10 min, followed by 40 cycles of amplification with 95 °C for 15 s then 60 °C for 1 min. Primer sequences used for human *MYOD* were (forward) 5'-TGCGCAACGCCATCCGCTATA-3' and (reverse) 5'-GGGGCCGCTGTAGTCCATCATG-3'; *MYOG* (forward) 5'-TGCTCAACCCCAACCAGCGG-3' and (reverse) 5'-GCATTCGCTGGG CACCCCT-3. T-test was used (*p* < 0.05) to determine statistical significance.

## Supplementary Materials

Supplementary materials can be accessed at: http://www.mdpi.com/1420-3049/20/05/8304/s1.
