# Supplementary material for: Culture Conditions Affect Expression of DUX4 in FSHD Myoblasts
Source: Molecules. 2015 May 8;20(5):8304–15. doi: 10.3390/molecules20058304 (PMC6272558; doi:10.3390/molecules20058304)
Supplement: Supplementary file 1 [file molecules-20-08304-s001.pdf]

Supplementary Materials

Table S1. cDNA synthesis strategies, PCR conditions and primer sequences, which have been used to amplify DUX4.

Publications	RNA Isolation	RNA Quantity (µg)	Primers Type	Reverse Transcriptases	Thermocycler	qPCR/PCR Reagents	PCR Conditions	DUX4 Amplification Primers
Snider *et al.*, 2010	Trizol reagent (Life Technologies), DNaseI digested followed by RNeasy (Qiagen) cleanup	1.5–2.0	Oligo(dT) (Life Technologies)	Superscript III (Life Technologies)	N/A	PCRX enhancer solution (Life Technologies) and Platinum Taq polymerase (Life Technologies)	**DUX4 detection:** primary and nested PCR: 94 °C for 5 min followed by 35 cycles of 94 °C for 30 s, 62 °C for 30 s, 68 °C for 2.5 min; **DUX4 quantification**: 95 °C for 5 min followed by 36 cycles of 95 °C for 30 s, 62 °C for 30 s, 68 °C for 1 min, and final extension at 68 °C for 5 min	**DUX4 detection:** F-5'-CCCCGAGCCAAAGC GAGGCCCTGCGAGCCT-3'; R-5'-GTAACT CTAATCCAGGTTTGCCTAGA-3'; nested F-5'-CGGCCCTGGCCCGGGAG ACGCGGCCCGC-3'; R-5'-TCTAATCCAGG TTTGCCTAGACAGC-3'; **DUX4 quantification:** F-5'-CAAGGGGTGCT TGCGCCACCCACGT-3'; R-5'-GGGGTGCG CACTGCGCGCAGGT-3'
Lemmers *et al.*, 2010	Macherey nagel total RNA isolation kit	0.5	Random hexamers	RevertAid H minus M-MuLV First strand kit (Fermentas)	MyiQ (Biorad)	SYBER green master mix (Stratagene)	95 °C for 3 min followed by 40 cycles of 95 °C for 10 s, 62 °C for 45 s	F-5'-CCCAGGTACCAGCAGACC-3'; R-5'-TCCAGGAGATGTAACTCTAATCCA-3'
Stadler *et al.*, 2012	RNeasy Plus kit (Qiagen); Qiashredder columns (Qiagen)	2.0	Random hexamers and oligo(dT)18 primers (Roche)	Transcriptor first-strand cDNA synthesis kit (Roche)	Lightcycler 480 (Roche)	SYBER green master mix (Roche)	95 °C for 3 min; 95 °C for10 s; 62 °C for 45 s	F-5'-CCCAGGTACCAGCAGACC-3'; R-5'-TCCAGGAGATGTAACTCTAATCCA-3'
Jones *et al.*, 2012	RNeasy Mini Kit (Qiagen). DNase I-treated on the column of the RNase-Free DNase Set (Qiagen)	1–1.5	Oligo(dT) 16 (Life Technologies)	Superscript III (Life Technologies)	N/A	Phusion Hot Start II High-Fidelity DNA Polymerase (Thermo Scientific)	98 °C for 2 min followed by 25 cycles of 98 °C for 15 s, 62 °C for 20 s, 72 °C for 1 min; nested PCR 98 °C for 2 min followed by 30 cycles of 98 °C for 15 s, 62 °C for 15 s, 72 °C for 50 s	F-5'CCCCGAGCCAAAGCGAGGCCCTGCG AGCCT-3'; R-5'-GTAACTCTAATCCAGGTTTGCCTAGA-3'; nested F-5'CGGCCCTGGCCCGGGAGA CGCGGCCCGC-3'; R-5'-TCTAATCCAGGTTTGCCTAGACAGC-3'

Table S1. *Cont.*

Publications	RNA Isolation	RNA Quantity (µg)	Primers Type	Reverse Transcriptases	Thermocycler	qPCR/PCR Reagents	PCR Conditions	DUX4 Amplification Primers
Cabianca *et al.*, 2012	Trizol reagent (Life Technologies) then purified with PureLink RNA MiniKit (Life Technologies) followed by DNaseI digestion	0.1–1	Oligo(dT)20 and random hexamers mix	Superscript III (Life Technologies)	N/A	SYBER Green ER qPCR kit (Life Technologies)		F-5'-GGACTCGAGACTCCGTTCAA-3'; R-5'-CCATCCTGAGTTCATTTTTGTG-3'
Block *et al.*, 2013	Trizol reagent (Life Technologies) followed by DNaseI digested and cleanup by phenol/chloroform	1.0	Oligo(dT) (Life Technologies)	Superscript III (Life Technologies)	N/A	PCRX enhancer system for GC rich sequences (Life Technologies) and Taq polymerase (New England Biolabs)	35 cycles of 94 °C for 30 s; 55 °C for 30 s; 68 °C for 120 s	F-5'-GCTGGAAGCACCCCTCAGCGAGGAA-3'; R-5'-GAATTCCATGGCGTCGTTTACTTT GACCAACAAGAA-3'
Present study	mirVANA kit (Life Technologies) followed by DNase I digestion and cleanup by mini elute RNA cleanup kit (Qiagen)	1.0	Oligo(dT)12-18 and random hexamers (Life Technologies)	Superscript II and Superscript III (Life Technologies)	2720 Thermal cycler (ABI)	GoTaq PCR mastermix (promega)	95 °C for 3 min followed by 40 cycles of 95 °C for 10 s, 62 °C for 45 s, and final extension at 72 °C for 10 min	F-5'-CCCAGGTACCAGCAGACC-3'; R-5'-TCCAGGAGATGTAACTCTAATCCA-3'
